# MXene-based novel nanocomposites doped SnO_2_ for boosting the performance of perovskite solar cells

**DOI:** 10.1038/s41598-024-64632-1

**Published:** 2024-06-25

**Authors:** T. F. Alhamada, M. A. Azmah Hanim, D. W. Jung, R. Saidur, A. A. Nuraini, W. Z. Wan Hasan, K. H. Tan, M. Mohamad Noh, M. A. M. Teridi

**Affiliations:** 1 Department of Mechanical and Manufacturing Engineering, Faculty of Engineering, Universiti Putra Malaysia, 43400 Serdang, Selangor Malaysia; 2 Department of Scientific Affairs, University Presidency, Northern Technical University, Mosul, 41001 Iraq; 3 Advanced Engineering Materials and Composites Research Center, (AEMC), Faculty of Engineering, Universiti Putra Malaysia, 43400 Serdang, Selangor Malaysia; 4 Faculty of Applied Energy System, Major of Mechanical Engineering, Jeju National University, 102 Jejudaehak-Ro, Jeju-Si, 63243 Republic of Korea; 5 Research Centre for Nano-Materials and Energy Technology (RCNMET), School of Engineering and Technology, Sunway University, 47500 Petaling Jaya,, Malaysia; 6 Department of Electrical and Electronic Engineering, Faculty of Engineering, UPM, 43400 Serdang, Malaysia; 7 Institute of Sustainable Energy (ISE), Universiti Tenaga Nasional (UNITEN), Jalan IKRAM-UNITEN, 43000 Kajang, Selangor Malaysia; 8 Solar Energy Research Institute, Universiti Kebangsaan Malaysia, 43600 Bangi, Selangor Malaysia; 9 School of Engineering, Lancaster University, Lancaster, LA1 4YW UK

**Keywords:** Nanocomposites, MXene, Perovskite solar cells, Hydrothermal method, Mechanical engineering, Engineering, Materials science, Nanoscience and technology, Optics and photonics, Optical materials and structures

## Abstract

Since being first published in 2018, the use of two-dimensional MXene in solar cells has attracted significant interest. This study presents, for the first time, the synthesis of an efficient hybrid electrocatalyst in the form of a nanocomposite (MXene/CoS)-SnO_2_ designed to function as a high-performance electron transfer layer (ETL). The study can be divided into three distinct parts. The first part involves the synthesis of single-layer Ti_3_C_2_T_x_ MXene nanosheets, followed by the preparation of a CoS solution. Subsequently, in the second part, the fabrication of MXene/CoS heterostructure nanocomposites is carried out, and a comprehensive characterization is conducted to evaluate the physical, structural, and optical properties. In the third part, the attention is on the crucial characterizations of the novel nanocomposite-electron transport layer (ETL) solution, significantly contributing to the evolution of perovskite solar cells. Upon optimising the composition, an exceptional power conversion efficiency of more than 17.69% is attained from 13.81% of the control devices with fill factor (FF), short-circuit current density (J_sc_), and open-circuit voltage (V_oc_) were 66.51%, 20.74 mA/cm^2^, and 1.282 V. Therefore, this PCE is 21.93% higher than the control device. The groundbreaking MXene/CoS (2 mg mL^−1^) strategy reported in this research represents a promising and innovative avenue for the realization of highly efficient perovskite solar cells.

## Introduction

The use of solar cells has grown dramatically in response to the growing demand for clean, renewable energy. The increasing demand for clean and renewable energy sources has focused much emphasis on the development of innovative nanomaterials for efficient solar cells^1–6^. Selective etching (extraction) of metals from carbides, including MAX phases, produced a multitude of these nanomaterials, including carbon nanotubes, graphene, carbide-derived carbons, and silicon carbide (SiC) nanoplatelets. Over 3000 papers have been written on the topic of MXenes by scientists worldwide in the ten years since its discovery. The Gogotsi group originally reported on MXenes as a family of important two-dimensional materials, layered carbides, and transition metal nitrides in 2011^7^. A solar cell is a device that uses the photovoltaic effect to directly convert light energy into electric energy. Interest in the application of two-dimensional MXene materials in photovoltaics has grown since the first study was conducted in 2018. Two-dimensional materials MXene exhibits potential in a variety of applications with its distinct characteristics. Recently, 2D transition metal have emerged as promising candidates for employment in Photovoltaic Solar Cells and supercapacitor applications^8–12^. The development of innovative materials for efficient solar cells has received a lot of interest due to the growing demand for clean and renewable energy resources. Morphology, conductivity, transparency, and termination groups are among the characteristics of MXenes^7^. In the last 6 years, there have been several studies in the literature that have used MXene materials in perovskite solar cells. The main aim of these studies is to enhance the performance of perovskite solar cells by incorporating MXene materials into the cell structure^13–18^. The goal of this research is to increase the photovoltaic performance of MXene/CoS-SnO_2_ nanocomposite/perovskite solar cell devices by giving them increased electrical conductivity and catalytic activity through the combination of high conductive MXene and high catalytic CoS catalyst.

## Methodology

**Figure 1:**
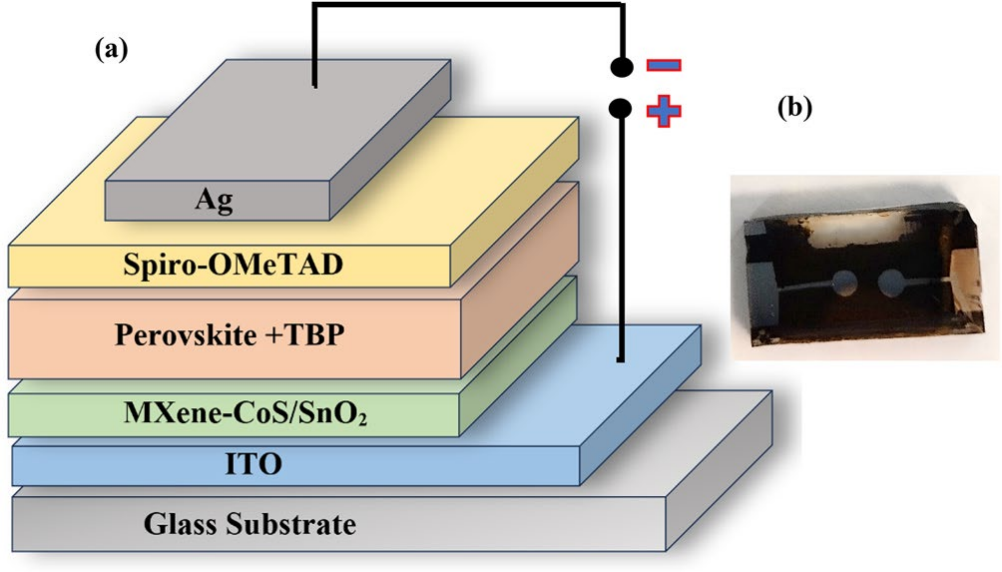
(**a**) Schematic of the PSCs structure and (**b**) perovskite solar cell device used in this study.

The novel nanocomposite has been developed for use in the perovskite solar cells device, characterizations of the nanocomposite-electron transport layer (ETL) solution that can play an essential role in perovskite solar cells as shown in Fig. 1, fabrication of the novel perovskite solar cell device that can use for light-harvesting for sustainable development.

### Materials

Ti_3_AlC_2_ powders (≤ 40 μm) was purchased from Y-CARBON, ltd., Kiev, Ukraine. ammonium hydrogen difluoride (NH_4_HF_2_; reagent grade 95%, Sigma Aldrich), sodium hydroxide (97% purity, pellets, Sigma Aldrich). Additionally, VWR Chemicals' 99.95% pure ethanol was used. Cetyltrimethylammonium bromide (CTAB) and all other Cobalt(II) Nitrate Hexahydrate (CoNO_3_⋅6H_2_O), Ammonium thiocyanate (CH_4_N_2_S), tin (iv) oxide-15% in H_2_O colloidal dispersion, PbI_2_ lead iodide, DMF dimethyl formamide, DMSO dimethyl sulfoxide, methylammonium bromide (MABr), Formamidinium iodide (FAI), methylammonium chloride (MACl), Spiro-OMeTAD, lithium bis[(trifluoromethyl)sulfonyl]imide salt (Li-TFSI, 99%), chlorobenzene (99%), acetonitrile (99.8%), 4-tert-Butylpyridine (TBP, 96%), and Indium tin oxide (coated glass slide) were bought from Sigma Aldrich/USA reagent and used without further purification.

### Fabrication of MXene/CoS heterostructure nanocomposite

**Figure 2:**
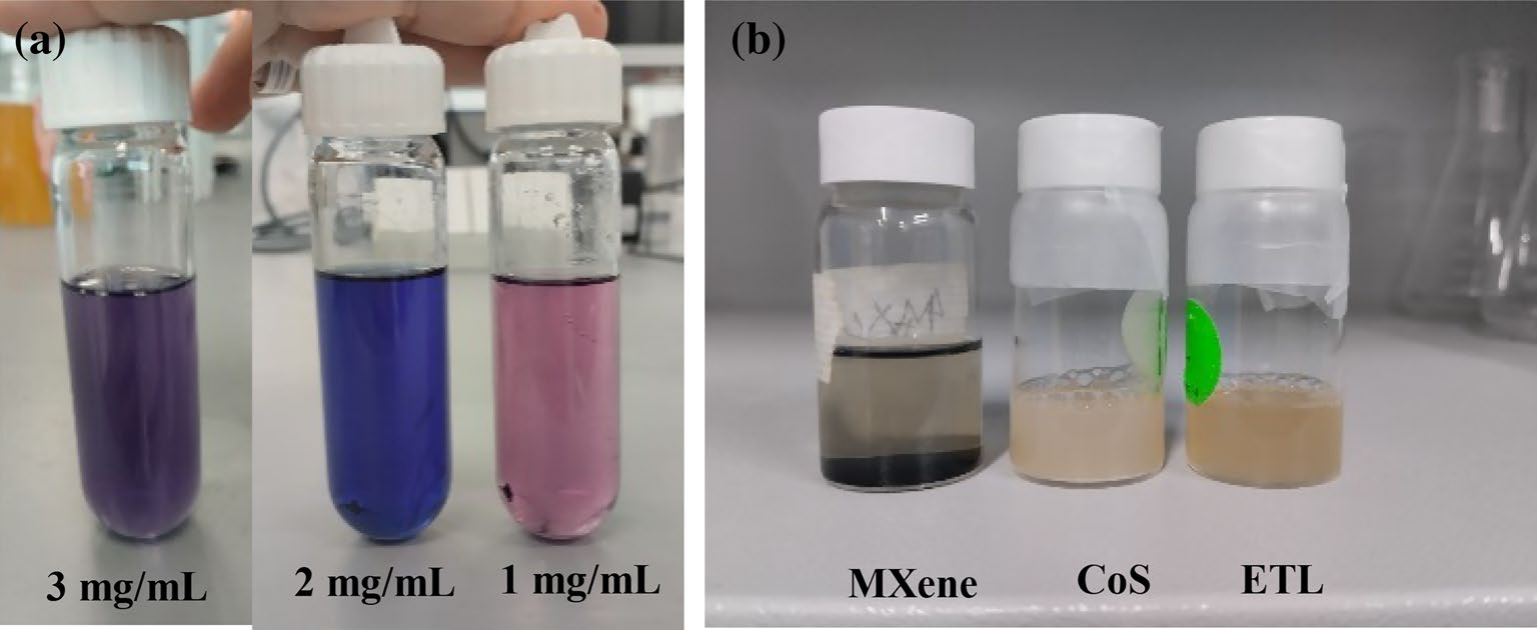
The optical photographs of the (**a**) MXene/CoS mixed solution with the different concentrations (mg/mL) and (**b**) MXene, CoS, nanocomposite/SnO_2_ (ETL).

We synthesized MXene/CoS heterostructure composites using the hydrothermal method, which are materials that consist of a layer of MXene combined with a layer of cobalt disulfide (CoS). MXene/CoS heterostructure nanocomposite was synthesized as shown in Fig. 2a, which are materials that consist of a layer of MXene (a type of two-dimensional transition metal carbide) combined with a layer of cobalt sulfide. MXene/CoS heterostructure composites (The combination of multiple heterojunctions together in a device) were synthesized using a simple and effective hydrothermal method. In the initial step, a mixture comprising 233 mg of CoNO_3_⋅6H_2_O and 122 mg of CH_4_N_2_S was introduced into 20 mL of absolute ethanol, followed by stirring for a duration of 30 min. Concurrently, a Ti_3_C_2_T_x_ colloidal suspension with a concentration of 0.4 mg mL^−1^ and containing 2 wt.% CTAB (Cetyltrimethylammonium bromide) was prepared., amounting to 5 mL. Then, the Ti_3_C_2_T_x_ MXene colloidal suspension (a mixture having particles of one component, with diameters between 10^−7^ and 10^−9^ (3.54 and 6.69 nm) meters, suspended in a continuous phase of another component) was slowly added into the above solution under magnetic stirring. The solution mixture was transferred into a Teflon-lined stainless steel autoclave, which had a capacity of 50 mL. Subsequently, the autoclave was positioned in a furnace and maintained at a constant temperature of 180 °C for a duration of 12 h. Following this, the Teflon-lined stainless-steel autoclave was left to cool down naturally to room temperature. Finally, the resulting MXene/CoS composite was obtained by subjecting it to centrifugation, followed by repetitive washing with Deionized water (DI water) and ethanol. The composite was then dried under vacuum at a temperature of 60 °C overnight. It is important to carefully follow the instructions of any synthesis method to ensure the production of high-quality MXene/CoS heterostructure composites^19,20^.

**Figure 3:**
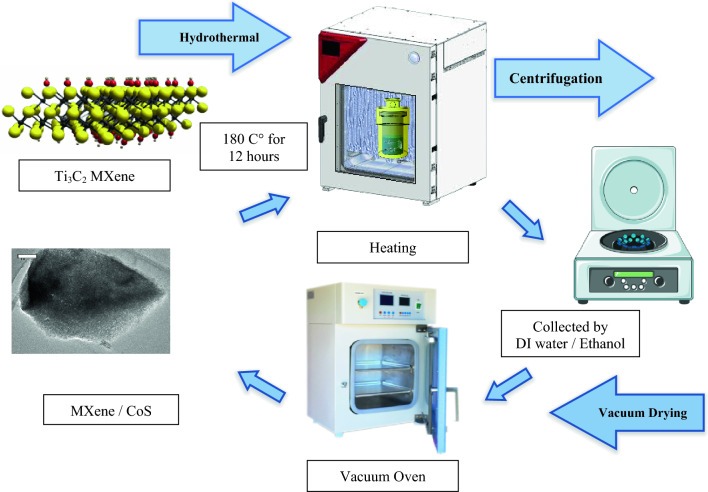
The process of forming MXene/CoS heterostructure composites is depicted in the schematic diagram.

For comparison purposes, pure CoS nanoparticles were also synthesized using similar steps described above, but without the presence of MXene solution. Furthermore, to achieve MXene/CoS composites with varying MXene content, high MXene content (sample Ti_3_C_2_T_x_-H/CoS) and low MXene content (sample Ti_3_C_2_T_x_-L/CoS) were prepared. This was accomplished by utilizing 2 mL and 7 mL of Ti_3_C_2_T_x_ colloidal suspension (0.4 mg mL^−1^) with 2 wt.% CTAB, respectively, the subsequent steps were carried out following the aforementioned procedures. Prior to usage, all solutions were filtered through a PTFE filter with a pore size of 0.2 μm. Figure 3 provides a visual representation of these processes.

### Preparation of the MXene/CoS heterostructure nanocomposite- SnO_2_ solution

The MXene/CoS heterostructure nanocomposite-SnO_2_ precursor solutions were prepared by mixing the diluted SnO_2_ solution with the powders of MXene/CoS heterostructure nanocomposite, followed by vigorous sonication for hours. The MXene/CoS heterostructure nanocomposite was weighed and dissolved in the SnO_2_ solution and maintained the concentration at 2 mg ml^−1^. The solvent was carefully added to the container with simultaneous continuous stirring to guarantee the thorough dissolution of the powders. Once the powders were entirely dissolved, resulting in a uniform solution, filtration was performed to eliminate any residual undissolved particles. Subsequently, the solution was carefully stored within a sealed container, maintaining it at the designated temperature, in anticipation of its forthcoming application. The preparation process of the electron transport layer (ETL) material, specifically the nanocomposite-SnO_2_ mixed solution, is intricately provided for a comprehensive understanding the optical photographs of the MXene/CoS mixed solution with the different concentrations. Figure 2a displays optical photographs of the Ti_3_C_2_T_x_/CoS nanocomposite-SnO2 mixed solution with varying concentrations (0,1,2, & 3 mg/mL). As the concentration of MXenes increases, the transparency of the mixed solution gradually decreases. Remarkably, as the concentration of MXene is progressively increased, a conspicuous reduction in the transparency of the mixed solution becomes evident, emphasizing the significant impact of MXene content on the optical properties. Furthermore, in Fig. 2b, a series of optical photographs exhibit nanocomposite/SnO_2_, CoS, MXene at varying samples.

### Preparation of the perovskite precursor solution

The preparation of a perovskite precursor solution involves combining the desired inorganic salt precursors in a solvent to achieve a uniform solution^21^. The perovskite layer was fabricated using a conventional two-step sequential deposition method^22,23^. Briefly, the PbI_2_ precursor solution was prepared by mixing PbI_2_ (600 mg) with DMF (0.95 mL) and DMSO (50 μL) at 60 °C for 4 h. After filtering, the perovskite solution was obtained. Next, a mixed solution containing 10 vol% TBP was stirred vigorously for at least one hour to ensure homogeneity. Subsequently, the coated layer was annealed at 150 °C for 15 min.

### Fabrication of the perovskite solar cells device

The ITO glasses were subjected to repeated washing steps in an ultrasonic bath containing detergent (Diluting 30 ml of Hellmanex III concentrate solution to 2% concentration will yield 1.5 L of cleaning solution), deionized water, acetone, and isopropanol solution. Afterward, they were dried using an N_2_ gun. The dried ITO glass was then treated in a UV ozone chamber for a duration of 30 min. Subsequently, the MXene/CoS heterostructure nanocomposite-SnO_2_ solution was separately spin-coated on the ITO substrates for 40 s at 3000 rpm. The ITO glasses, now coated with an electron transfer layer (the average thickness is ~ 50 nm), were placed on a hot plate, maintaining a constant temperature of 150 °C for 15 min, followed by natural cooling to room temperature. Next, The PbI_2_ precursor was then spin-coated onto the ETL at 1500 rpm for 30 s and annealed at 70 °C for 1 min. Afterward, a solution containing FAI, MACl, and MABr precursors (60 mg of FAI, 6 mg of MACl, and 6 mg of MABr dissolved in 1 mL of IPA) was applied using spin-coating onto the PbI_2_ layer at 1300 rpm for 30 s. During the last 10 s of the spin-coating process, the antisolvent chlorobenzene (CB) solution was applied. After applying the electron transfer layer and perovskite layer (275–325 nm) onto the ITO substrates, they were returned to the hot plate and maintained at a constant temperature of 150 °C for a duration of 15 min. Subsequently, the substrates were left to gradually cool down to room temperature. Once cooled, the Spiro-OMeTAD, which was dissolved in a CB solution, was employed for spin-coating onto the perovskite layer. The HTL (the average thickness is ~ 250 nm) was prepared by spin-coating a mixture solution containing 23 μL of Li-TFSI solution (520 mg of Li-TSFI in 1 mL of acetonitrile), 36 μL of TBP, 90 mg of spiro-OMeTAD, and 1 mL of CB at 3000 rpm for 30 s. Subsequently, the devices were placed in a clean box under at 25 °C for 24 h to oxidize the surfaces. Finally, the unfinished installation was transferred into an airtight environment for the deposition of a 100 nm layer of silver (Ag) via thermal evaporation at a base pressure of 4.0 × 10^−4^ Pa, serving as the back contact electrode. The active area of the devices was maintained at 0.07 cm^2^^24,25^.

## Results and discussion

This research focuses on the photovoltaic performance of a novel category of nanocomposites infused with MXene nanoparticles in three different concentrations, as well as their preparation, characterization, configurations, materials, and fabrication methods. Therefore, the remarkable features of these nanocomposites, characterized by their high electrical conductivity, tunable band gaps, enhanced electronic conductivity, excellent chemical stability, and the ability to accommodate ion intercalation, establish them as an exceedingly promising choice for a myriad of applications.

The results are presented all the Compounds and perovskite solar cell devices were characterised by HRTEM, SEM, EDX, CV, EIS, XRD, UV–Vis, and solar simulator to examine their structural, physical, and optical properties.

### Structural & morphology characterization

With the structural characterization, SEM and XRD analyses were conducted, to verify the structure of the synthesized Ti_3_C_2_T_x_ /CoS, CoS, and Ti_3_C_2_T_x_. These observations suggest that the Ti_3_C_2_T_x_ /CoS, Ti_3_C_2_T_x_, and CoS samples have undergone satisfactory crystallization during synthesis, thereby confirming their crystalline structures. The FESEM images shows that tiny spherical CoS nanoparticles are evenly spread over the Ti_3_C_2_T_x_ flakes with a diameter range of 3.54–6.69 nm. The SEM images indicate a structure of Ti_3_C_2_T_x_, which enables the formation of effective sites, leading to uniform deposition of CoS nanoparticles on the conductive MXene sheets. The SEM images also show the MXene/CoS composite, revealing the uniform attachment of CoS nanoparticles to the Ti_3_C_2_T_x_ layers. Based on the morphological characterization, it's clear that MXene has distinct and well-defined lattice fringes with a lattice spacing of 4.22 nm. The morphology is thin and electron-transparent, which suggests a single-layer structure. The combination of layered MXene with small CoS nanoparticles (3.54–6.69 nm) provides numerous catalytic active sites. Moreover, the improved mesoporous morphology of the Ti_3_C_2_T_x_ /CoS composite allows for a high specific surface area and facilitates rapid electron transfer channels. Therefore, the Ti_3_C_2_T_x_ /CoS nanocomposite has the potential to be an excellent material that can enhance the photovoltaic performance of the electron transport layer (ETL). Elemental map imaging and spatial distribution of all elements were confirmed using energy dispersive X-ray spectroscopy (EDX) with an Oxford Instrument, for details see supplementary information.

### Ultraviolet–visible absorption spectrum characterization

The photocatalytic activity can be measured by monitoring the reaction rate using techniques such as Ultraviolet–visible (UV–Vis) spectroscopy. One important use of phase change materials is the transformation of abundant solar energy into useful energy. It enables the possibility of storing energy during sunlight exposure and utilizing it during the night. This energy storage method, known as adsorptive energy storage, can be evaluated by employing UV–Vis spectrometry at various wavelengths.

**Figure 4:**
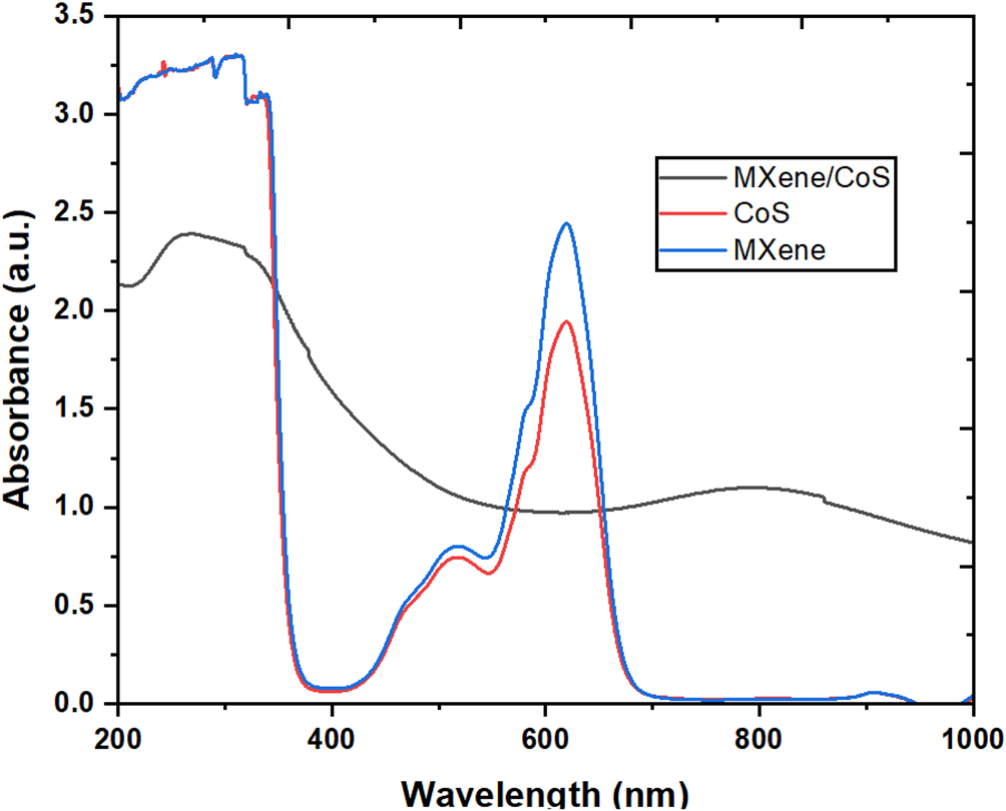
UV–Vis spectra of the MXene, CoS and MXene/CoS samples.

To overcome this challenge and enhance the photoelectrochemical performance of photocatalysts, one approach is to construct heterostructures. Figure 4 presents the results of ultraviolet and visible (UV–Vis) spectroscopy conducted on delaminated Ti_3_C_2_T_x_ MXene (at a concentration of 0.05 mg/mL), CoS, and MXene/CoS nanocomposite.

**Figure 5:**
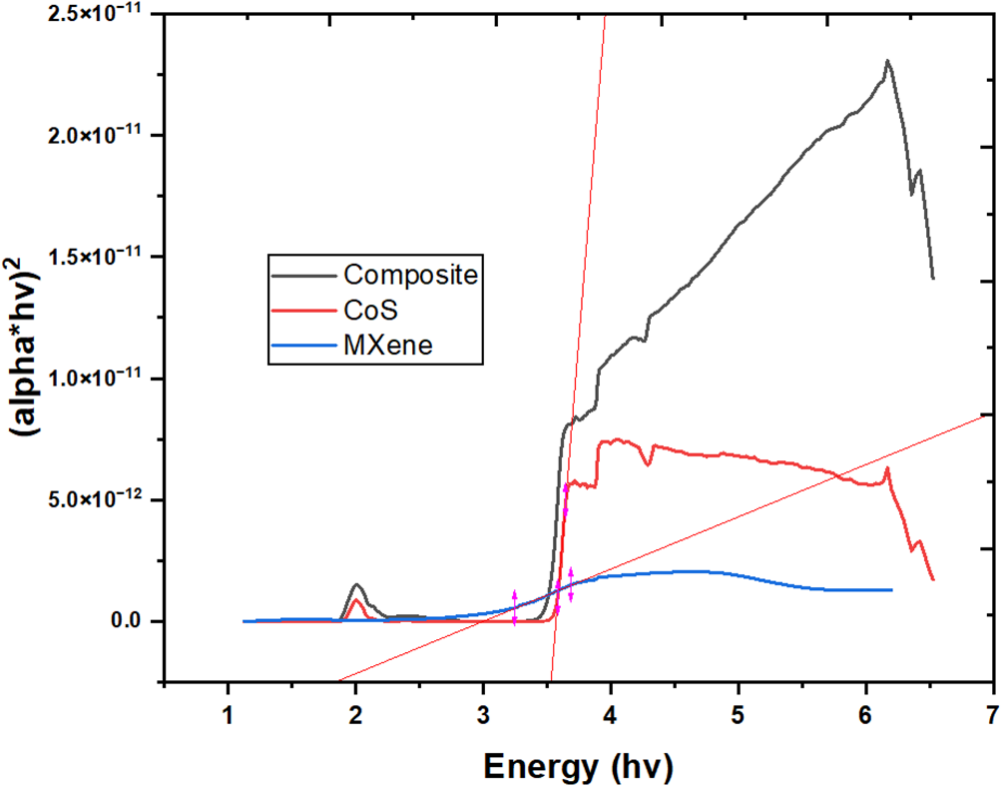
Band gaps of nanocomposite, MXene, and CoS estimated by (alpha*hυ)^2^vs. photon energy curve.

In the dilute aqueous medium, MXene colloid displays a broad UV absorption spectrum with peaks observed at 225 and 275 nm^26,27^. Based on the UV–Vis characterization, it's clear that the delaminated MXene exhibits significant absorption in the UV region spanning from 225 to 365 nm. This outcome is anticipated due to the functionalization of the MXene surface with different groups following the removal of the A-element from its precursor ternary transition metal carbide, also known as the MAX phase, during the synthesis process. The absorption peaks of CoS and MXene/CoS nanocomposite appear at wavelengths ranging from 210 to 358, and 553 to 657 nm, respectively. The nanocomposite exhibits higher photoelectrochemical properties compared with pure CoS nano-powder. The improved photoelectrochemical efficiency can be ascribed to the augmentation of light absorption and the decrease in the recombination rate of photogenerated electron–hole pairs, which stem from the creation of the heterostructure. Interestingly, the results indicate that the nanocomposite expands the absorption spectra. Tauc's plot analysis^28^, as illustrated in Fig. 5, estimates the band gaps of MXene and CoS to be 1.86 eV and 3.54 eV, respectively. Additionally, the nanocomposite reduces the band offset at the interface between the nanocomposite-SnO_2_ and the perovskite layers.

### CV & EIS characterization of the MXene, CoS and MXene/CoS based ETL

**Figure 6:**
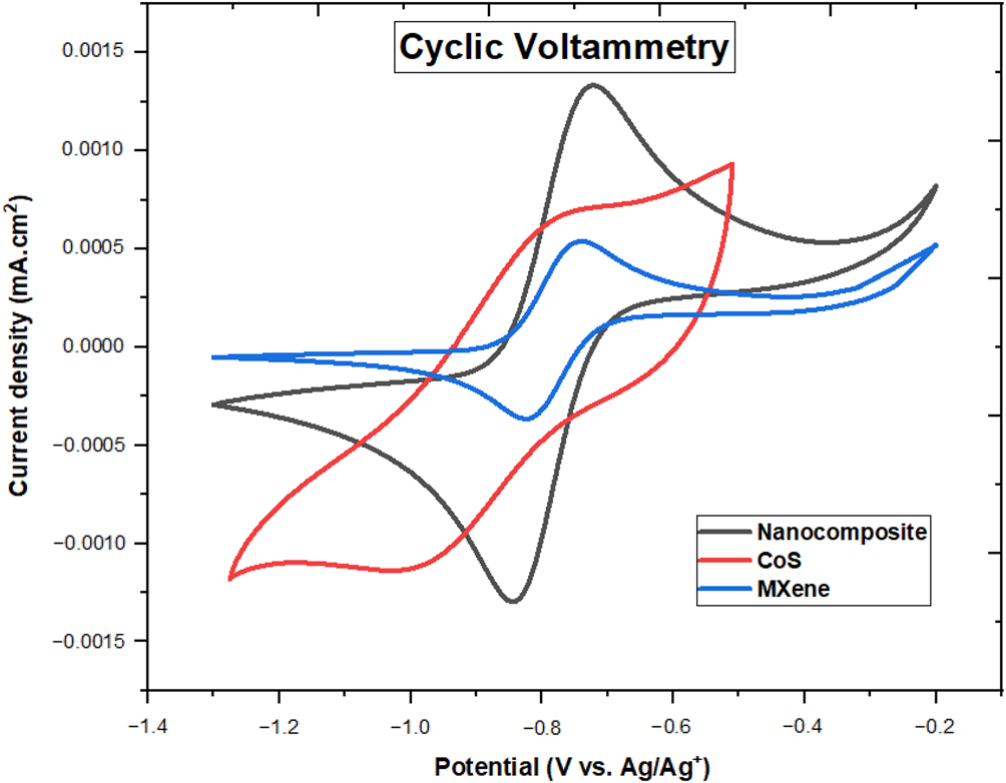
Shows the cyclic voltammetry (CV) profiles of the pure MXene, CoS and MXene/CoS.

To verify the proposed new solar cell layer that contains an MXene-based nanocomposite, several strategies can be used for the evaluation and analysis of the measurement results. Electrochemical techniques, such as electrochemical impedance spectroscopy (EIS), and cyclic voltammetry (CV) can be used to evaluate the electrochemical performance of the new solar cell layer and its components. Also, they can provide valuable information about the properties and performance of the proposed new solar cell layer and help to verify its potential for use in practical applications^29^. The presence of a higher reduction current density peak and a lower peak-to-peak separation (Epp) value serves as an indication of the strong catalytic reduction capability of Sx^−2^ at the ETL layer^30^. As depicted in Fig. 6, the MXene/CoS-ETL exhibits a higher reduction current density compared to the bare CoS-ETL and Ti_3_C_2_T_x_-ETL. Furthermore, the nanocomposite material exhibits an increase in the area under the CV curve and a transition from asymmetrical to symmetrical shape. This observation indicates a positive impact on the electrocatalytic activity.

The Nyquist plots of Ti_3_C_2_T_x_, CoS, and Ti_3_C_2_T_x_ /CoS-based ETL are shown along with the equivalent circuit fitting parameters, including series resistance (R_S_), charge transfer resistance (R_CT_), and C_1_ corresponding to the capacitance. In the symmetric dummy cell, the Ti_3_C_2_T_x_/CoS counter electrode (CE) displayed the smallest values for R_S_ (5.125 ohms), R_CT_ (1048 ohms), and C_1_ (4.925e^−3^ F). The lower R_S_, R_CT_ and higher C_1_ values suggest that PSCs with MXene/CoS CE display the better performance^31^. The origin of R_S_ is associated with the resistance of the ITO substrates, while R_CT_ is related to the resistance at the interface between the ETL and the perovskite layer. In this study, the semicircle observed in the high-frequency range of the EIS spectra offers valuable insights into the charge carrier recombination process, specifically the interfacial recombination and charge transfer processes from the ETL to the ITO electrodes. The rapid nature of the charge transfer process prevents its separation from the interfacial recombination. On the other hand, the semicircle observed at relatively lower frequencies corresponds to ion migration processes within the electron transfer layer^32^.

**Figure 7:**
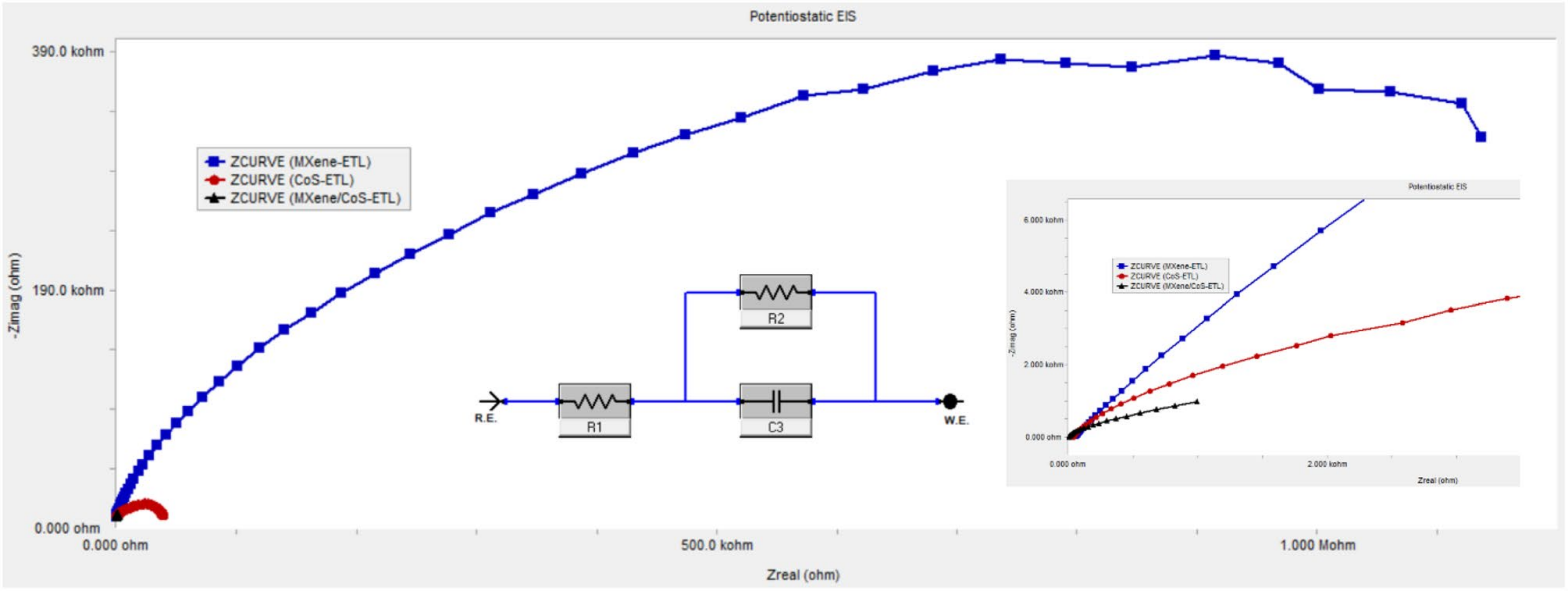
Presents the Nyquist plot curves of symmetric dummy cells equipped with Ti_3_C_2_T_x_, CoS, and Ti_3_C_2_T_x_/CoS -based ETL. The inset within the figure provides a closer look at the EIS curves and displays the equivalent circuit employed for fitting the EIS data.

Figure 7 show the Bode and Nyquist plots progression with different electron transport layers. In the high-frequency region of the EIS spectra, a single semicircle was observed, corresponding to a single peak in the bode plot.

The parameters of EIS from the semicircle Nyquist plot are calculated and summarized in (Table 1). The rise in resistance suggests a decrease in charge recombination at the interface between CoS and ITO. Due to the limited rate of carrier recombination at the CoS/ITO junction, it is rational to expect that the CoS electron transport layer (ETL) exhibits both superior Jsc and Voc in comparison to those of the MXene-ETL^33^.

**Table 1:** Electrochemical parameters fabricated with the different ETL calculated from EIS analysis.

Layers	R_S_ (ohm)	R_CT_ (ohm)	C_1_ (F)
MXene/CoS-ETL	5.125	1.048e3	4.925e-3
CoS-ETL	44.57	16.54e3	497.0e-9
MXene-ETL	64.45	353.5e3	139.1e-9

Furthermore, the nanocomposite material exhibits an increase in the area under the CV curve and a transition from asymmetrical to symmetrical shape. the increased surface area resulting from doping facilitates the availability of more electrochemically favorable and active sites, thereby promoting higher conductivity. Electrochemical impedance spectroscopy (EIS), can be used to evaluate the electrochemical performance of the new solar cell layer and its components. The R_2_ of the MXene/CoS-ETL was decreased, this phenomenon can be attributed to the enhanced electron back transfer process, resulting in rapid charge recombination events. The presence of high series resistance in a device often results in a poor fill factor, which is a measure of its performance. Consequently, the justification for carbon-based perovskite solar cells (PSCs) exhibiting a lower fill factor is attributed to the inherent high series resistance associated with carbon-based materials. This resistance adversely affects the device's ability to efficiently collect and transport charge carriers, leading to reduced fill factor values^34,35^. It is also revealed that with the MXene/CoS-ETL, the R_CT_ was decreased, this phenomenon can be attributed to the enhanced electron back transfer process, resulting in rapid charge recombination events.

### The perovskite solar cells device characterization

Figure 1 depicts the manufacturing schematic of perovskite solar cells (PSCs) to confirm the performance boost caused by the insertion of the electron transport layer (ETL). The choice and qualities of the ETL can have a significant influence on the overall performance and efficiency of the produced solar cell. The ETL supports efficient charge movement, reduces recombination losses, improves electron extraction from the photoactive layer, and improves the overall electrical efficiency of the solar cell. Thus, careful attention and optimisation of the ETL are required to get high-performance solar cells.

**Figure 8:**
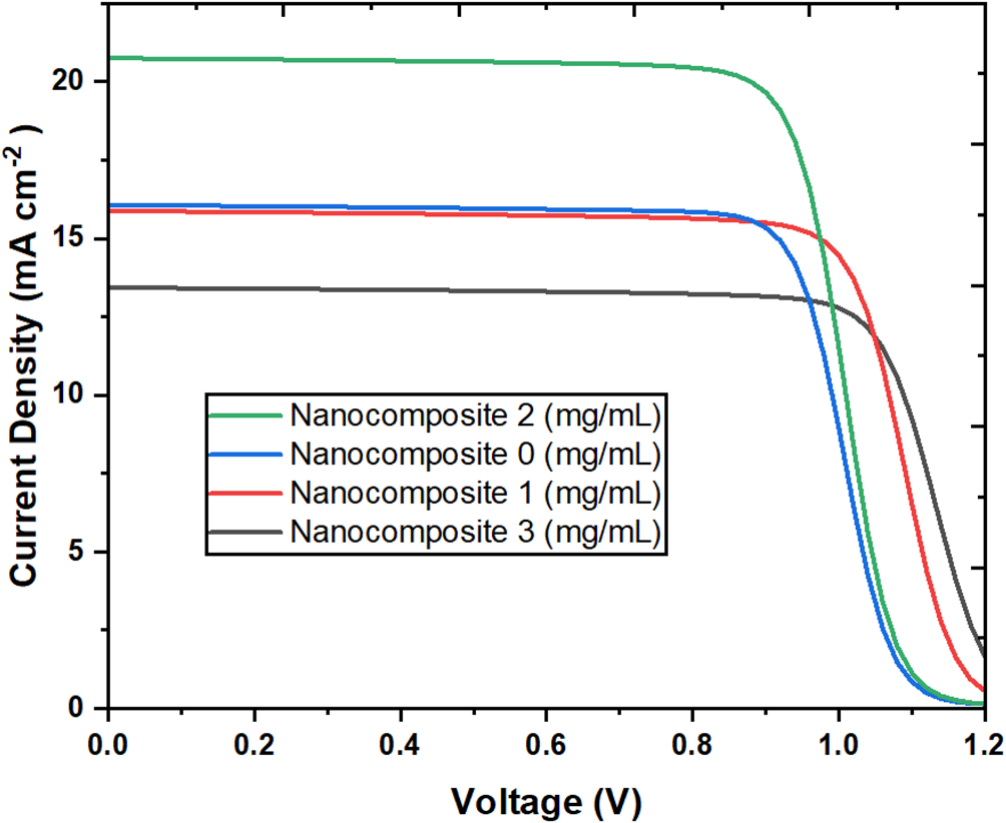
Current density of the champion devices based on various MXene/CoS nanocomposites.

The top-performing MXene-CoS/SnO_2_-based PSC was named the champion device, and its performance was determined by the concentration of MXene-CoS/SnO_2_. Figure 8 depicts the PCEs of PSCs at different concentrations. The best concentration, 2 mg/ml, resulted in the champion device with an enhanced PCE of 17.69%, a fill factor (FF) of 66.51%, an open-circuit voltage (V_oc_) of 1.282 V, and a short-circuit current density (J_sc_) of 20.74 mA/cm^2^. Understanding and analysing these edge effects in the J-V curves of champion devices made from various MXene/CoS nanocomposites is critical for optimising device performance, identifying limitations, and guiding improvements in materials, device fabrication, or device engineering to improve solar cell efficiency and stability. Analysing the J-V curves around the open-circuit voltage (V_oc_) has helped determine the device's efficiency, stability, and limitations. This research provides critical insights that will affect material optimisation, manufacturing methods, and device engineering, ultimately enhancing the overall performance of solar cells. Furthermore, improved characterisation techniques and theoretical modelling can help to clarify the precise elements that influence these curve properties.

**Figure 9:**
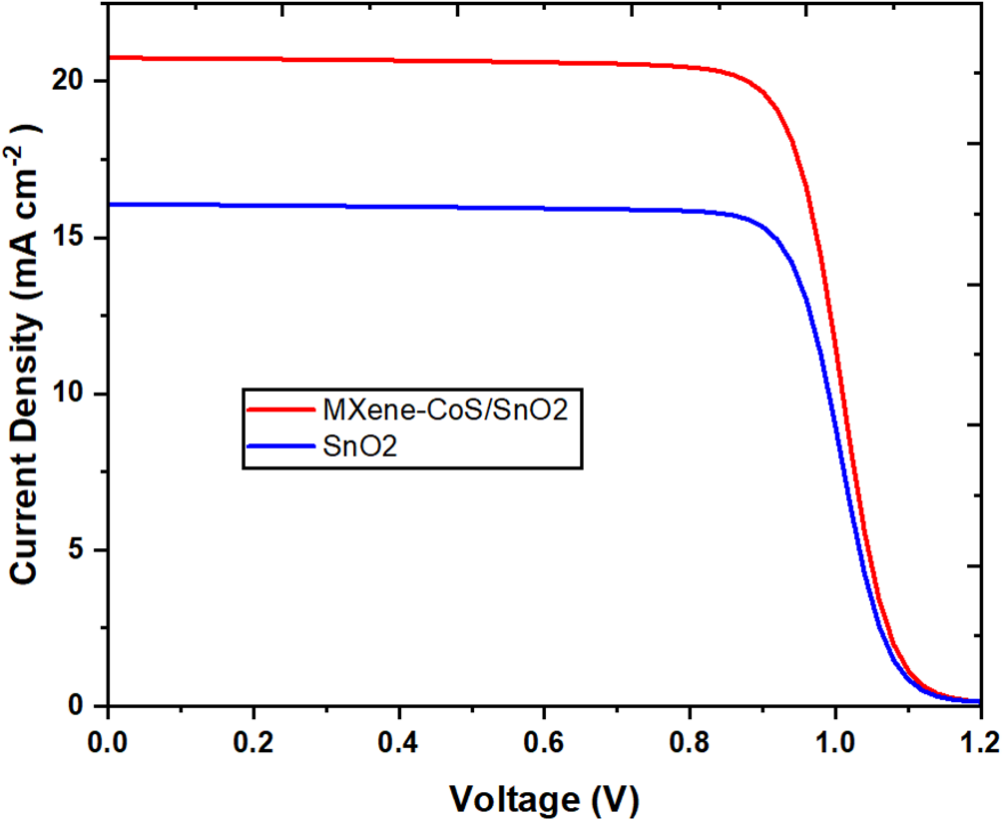
Illustrates the current density versus voltage (J-V) curves for PSCs based on the SnO_2_ and MXene-CoS/SnO_2_ ETLs under 1 sun (AM 1.5 G) illumination.

The enhanced FF value can be linked to an improved contact interface between the perovskite and ETL films, contributing to a more balanced charge transport and decreased accumulation of interface charges. Figure 9 illustrates the current density versus voltage (J-V) profiles of the perovskite solar cells (PSCs) deposited on SnO_2_ ETL (depicted by the grey curve) and MXene-CoS/SnO_2_ ETL (depicted by the blue curve) under 1 sun (AM 1.5 G) illumination. The power conversion efficiency (PCE) rose from 13.81% for the control PSC based on pure SnO_2_ ETL to 17.69% (as seen in Table 2). The open-circuit voltage (V_oc_) in perovskite solar cells can decline due to material defects, perovskite degradation caused by environmental factors, hysteresis effects, subpar interface quality, doping inconsistencies, and material degradation over time. Overcoming these challenges is essential to boosting V_oc_ and enhancing the overall performance of the device. The leading PSCs utilizing MXene-CoS/SnO_2_ ETL showcased notably improved performance, resulting in a noteworthy PCE. This enhancement in performance can be attributed to the amplified J_sc_ and FF, which were facilitated by diminished charge recombination and an enhanced quality of the perovskite film, respectively.

**Table 2:** Photovoltaic parameters of PSCs with different amount of MXene based nanocomposite.

MXene concentration	FF (%)	J_SC_ (mA cm^−2^)	I_MAX_ (mA cm^−2^)	V_OC_ (V)	V_MAX_ (V)	PCE (%)
0 (mg/mL)	65.78	16.072	15.320	1.306	0.901	13.81
1 (mg/mL)	77.88	15.877	14.998	1.229	0.974	14.61
2 (mg/mL)	66.51	20.749	19.700	1.282	0.898	17.69
3 (mg/mL)	77.42	13.432	12.671	1.232	1.011	12.81

To discover the appropriate settings and examine how physical variables impact model performance, numerical simulation of a photovoltaic device is required. The simulation approach is validated by comparing its results to experimental data. In this work, the SCAPS-1D programme is used to simulate the device, comprehensively investigating the effect of various structural characteristics of the electron transport layer (ETL) on the solar cell's performance.

**Figure 10:**
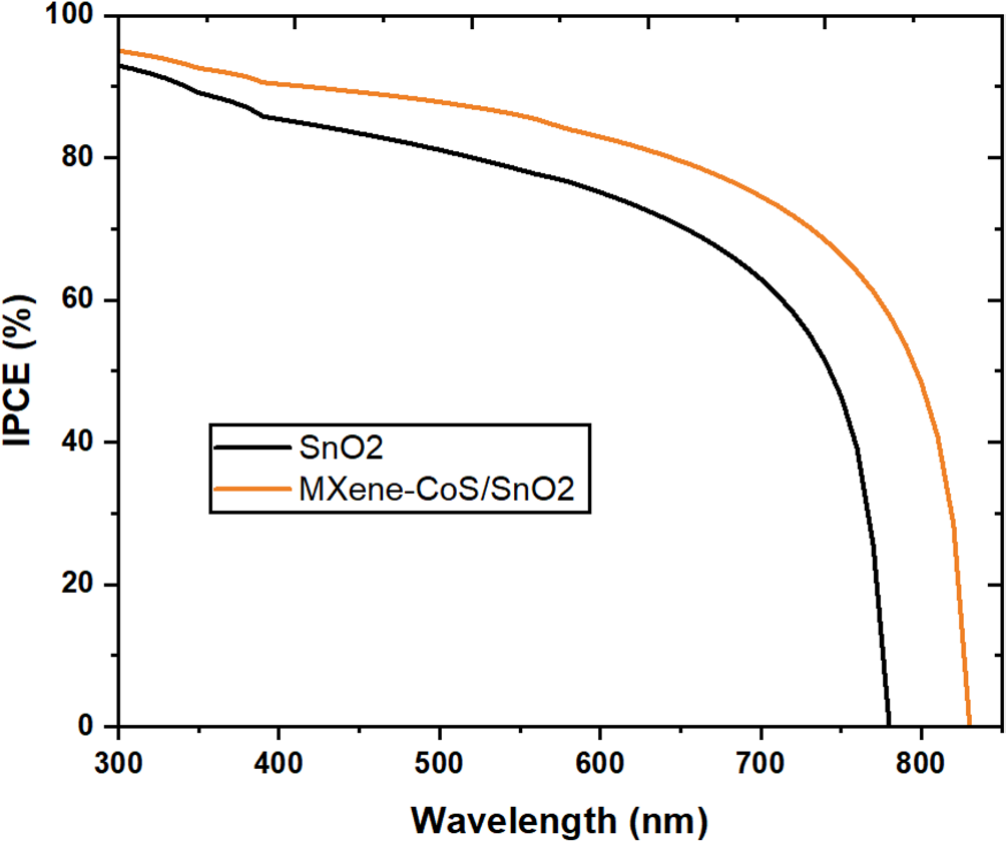
IPCE of the SnO_2_, and MXene-CoS/SnO_2_.

The suggested device structure is ITO/MXene-CoS/SnO_2_/Perovskite/Spiro-OMeTAD/Ag, with MXene-based innovative nanocomposites-doped SnO_2_ serving as the ETL for the next generation solar cell devices. The purpose of this numerical simulation is to show how solar cell efficiency may be increased by properly capturing incident light on its surface and optimising its performance. The superiority of PSCs with CoS, MXene, and MXene/CoS over SnO_2_ is further confirmed through the incident photon-to-current efficiency (IPCE) measurement, as shown in Fig. 10. The MXene/CoS exhibits better IPCE characteristics than SnO_2_, providing additional evidence of its improved performance as an ETL for the solar cell. The extended wavelengths beyond 780 nm in the IPCE of SnO_2_ and MXene-CoS/SnO_2_ indicate additional absorption of lower-energy photons. This happens due to factors like sub-bandgap absorption, quantum effects in nanoscale structures, and impurity-induced energy levels within the bandgap. Comparing experimental IPCE data and theoretical bandgap calculations can reveal the extent of this extended absorption.

## Conclusions

In conclusion, novel nanocomposites have been successfully developed using MXene and CoS for perovskite solar cells. The highly electrocatalytic MXene/CoS nanocomposite, used as additives to the SnO_2_ ETL, has been successfully synthesized through the hydrothermal technique and exfoliation process. The results also demonstrate the nanocomposite's ability to broaden the absorption spectra. Utilizing Tauc's plot, the band gaps of MXene and CoS were estimated to be 1.86 eV and 3.54 eV, respectively. X-ray diffraction (XRD) analysis confirms the high crystalline quality of the solar cell layer samples, evident by the presence of peaks at 5.6°, 25.16°, and 47.4° corresponding to the (002), (004), and (110) crystal planes of Ti_3_C_2_T_x_. Additionally, the enhanced photovoltaic performance of the nanocomposites can be attributed to the conductive MXene nanosheets acting as a scaffold, facilitating the growth of small CoS nanoparticles and generating abundant catalytic active sites. These nanocomposites also exhibit excellent permeability, remarkable charge transfer, and efficient ion diffusion.

Based on the results obtained, it can be concluded that using Novel MXene/CoS nanocomposites-Doped in SnO_2_ as ETL is essential in determining the characteristics and properties of the solar cell layers. The nanocomposites exhibit a remarkable electrochemical performance, with R_1_ (5.125 Ω), R_2_ (1.048e^−3^ Ω) and C_3_ (4.925e^−3^ F) values, respectively. These values are considerably better than those achieved with ETLs containing CoS and MXene alone. Additionally, the novel nanocomposites (MXene reinforced with CoS)-doped SnO_2_ as the ETL demonstrate noticeable growth of SnO_2_ grains. HAADF STEM analysis proved that the incorporation of the nanocomposite led to an increase in the lattice spacing facets of SnO_2_, ultimately improving the nanocomposite as ETL additive as contrasted with the SnO_2_ (ETL) film under control. In summary, our investigation has confirmed that incorporating MXene/CoS nanocomposite (2 mg mL^−1^) as additives to the SnO_2_ electron transport layer (ETL) can significantly increase the efficiency of PSCs. With the optimized composition, the solar cell's power conversion efficiency (PCE) measured a remarkable 17.69%., a significant improvement over the control devices with a PCE of 13.81%. The short-circuit current density (J_sc_), open-circuit voltage (V_oc_), and fill factor (FF) are computed at 66.51%, 1.282 V, and 20.74 mA/cm^2^, respectively. This design philosophy results in a 21.93% higher PCE compared to the control device. The outstanding potential of this design lies in its ability to promote electron transport efficiency and improve the quality of the perovskite layer through the introduction of 4-tert-butylpyridine as an additive. This manipulation of the PbI_2_ morphology enables the fabrication of efficient PSCs in ambient air with relative humidity ranging from 30 to 40%. Overall, this approach represents a way of opting for highly efficient perovskite solar cells.

### Supplementary Information

Supplementary Information.

## Data Availability

All data generated or analysed during this study are included in this published article and its supplementary information files.

## References

[1] Ma YMFZQAMZX (2018). Efficient ternary polymer solar cells with two well-compatible donors and one ultranarrow bandgap nonfullerene acceptor. Adv. Energy Mater..

[2] Zhao CZHKLGD (2015). High-performance Ta2O5/Al-doped Ag electrode for resonant light harvesting in efficient organic solar cells. Adv. Energy Mater..

[3] Wan HLSRRHYJZ (2020). Interfacial engineering in lead-free tin-based perovskite solar cells. J. Energy Chem..

[4] Zhao XHLCCDJLY (2019). Effects of selenization conditions on microstructure evolution in solution processed Cu2ZnSn(S, Se)4 solar cells. Sol. Energy Mater. Sol. Cells.

[5] Zhang YQJZJHS (2018). Over 14% efficiency in polymer solar cells enabled by a chlorinated polymer donor. Adv. Mater..

[6] Yin L (2021). MXenes for solar cells. Nanomicro. Lett..

[7] Gogotsi Y, Anasori B (2019). The rise of MXenes. ACS Nano.

[8] Kumar N (2022). One-step fragmentation of a 2D MXene across the fine 1D MnO2 surface and its supercapacitance. CrystEngComm.

[9] Shetty M (2023). Rapid single pot synthesis of hierarchical Bi2WO6 microspheres/RGO nanocomposite and its application in energy storage: A supercritical water approach. J. Energy Storage.

[10] Shetty M (2021). One-pot supercritical water synthesis of Bi2MoO6-RGO 2D heterostructure as anodes for Li-ion batteries. Ceram Int..

[11] Chetana S (2023). A facile supercritical fluid synthesis of cobalt sulfide integrated with MXene and PANI/PEDOT nanocomposites as electrode material for supercapacitor applications. FlatChem.

[12] Kumar N (2023). Blending of a 3D cloud-like morphology with a 1D structure in a VO2/MXene nanocomposite to enhance the charge storage capability. J. Mater. Chem. C Mater..

[13] Qureshi AA, Javed S, Akram MA, Schmidt-Mende L, Fakharuddin A (2023). Solvent-assisted crystallization of an α-Fe2O3electron transport layer for efficient and stable perovskite solar cells featuring negligible hysteresis. ACS Omega.

[14] Qureshi AA, Javed S, Adnan M, Jamshaid M, AftabAkram M, Ali U (2023). Strategic optimization of annealing parameters for efficient and low hysteresis triple cation perovskite solar cell. ChemistrySelect.

[15] Ali U, Qureshi AA, Javed S, ur Rehman G, Akram MA (2023). Graphene oxide incorporation in Ag-doped ZnO nanocomposite as efficient electron extraction material for planar perovskite solar cells. Results Opt..

[16] Qureshi AA, Javed S, Fakharuddin A, Akram MA, Schmidt-Mende L (2023). Low-temperature processed natural hematite as an electron extraction layer for efficient and stable perovskite solar cells. Surfaces Interfaces.

[17] Qureshi AA, Schütz ER, Javed S, Schmidt-Mende L, Fakharuddin A (2023). An Fe3O4 based hole transport bilayer for efficient and stable perovskite solar cells. Energy Adv..

[18] Ali U, Javed S, Qureshi AA, Akram MA (2023). Interfacial engineering of a PCBM/AZO electron transport bilayer for efficient and stable inverted perovskite solar cells. ChemNanoMat.

[19] Chen X, Zhuang Y, Shen Q, Cao X, Yang W, Yang P (2021). In situ synthesis of Ti3C2Tx MXene/CoS nanocomposite as high performance counter electrode materials for quantum dot-sensitized solar cells. Solar Energy.

[20] Liu Z (2022). MXene/CoS heterostructures self-assembled through electrostatic interaction as superior microwave absorber. J. Alloys Compd..

[21] Liu D (2018). Aqueous-containing precursor solutions for efficient perovskite solar cells. Adv. Sci..

[22] Jiang Q (2017). Planar-structure perovskite solar cells with efficiency beyond 21%. Adv. Mater..

[23] Chouhan AS, Jasti NP, Avasthi S (2018). Effect of interface defect density on performance of perovskite solar cell: Correlation of simulation and experiment. Mater. Lett..

[24] Mohamad Noh MF (2022). Facile tuning of PbI2 porosity via additive engineering for humid air processable perovskite solar cells. Electrochim Acta.

[25] Zheng H (2021). Controlling the defect density of perovskite films by MXene/SnO_2_ hybrid electron transport layers for efficient and stable photovoltaics. J. Phys. Chem. C.

[26] Satheeshkumar E, Makaryan T, Melikyan A, Minassian H, Gogotsi Y, Yoshimura M (2016). One-step solution processing of Ag, Au and Pd@MXene hybrids for SERS. Sci. Rep..

[27] Bakthavatchalam B, Habib K, Saidur R, Aslfattahi N, Rashedi A (2020). Investigation of electrical conductivity, optical property, and stability of 2D MXene nanofluid containing ionic liquids. Appl. Sci..

[28] He G, Zhang Y, He Q (2019). MoS2/CdS heterostructure for enhanced photoelectrochemical performance under visible light. Catalysts.

[29] Xiong XLZBSLD (2018). Recent advances in layered Ti3C2T MXene for electrochemical energy storage. Small.

[30] Ramírez D (2023). Hybrid potentiodynamic/potentiostatic electrodeposition of thin and compact tin dioxide on indium tin oxide electrodes. Electrochim Acta.

[31] Kuklin SA, Safronov SV, Khakina EA, Buyanovskaya AG, Frolova LA, Troshin PA (2023). New perylene diimide electron acceptors for organic electronics: Synthesis, optoelectronic properties and performance in perovskite solar cells. Mendeleev Commun..

[32] Li D (2017). Amino-functionalized conjugated polymer electron transport layers enhance the UV-photostability of planar heterojunction perovskite solar cells. Chem. Sci..

[33] Wang JF (2017). Surface engineering of perovskite films for efficient solar cells. Sci. Rep..

[34] Dileep R (2019). Room-temperature curable carbon cathode for hole-conductor free perovskite solar cells. Solar Energy.

[35] Veerappan G, Bojan K, Rhee SW (2012). Amorphous carbon as a flexible counter electrode for low cost and efficient dye sensitized solar cell. Renew. Energy.

