# Supplementary material for: MXene-based novel nanocomposites doped SnO2 for boosting the performance of perovskite solar cells
Source: Sci Rep. 2024 Jun 25;14:14638. doi: 10.1038/s41598-024-64632-1 (PMC11199647; doi:10.1038/s41598-024-64632-1)
Supplement: Supplementary file 1 — Supplementary Information. [file 41598_2024_64632_MOESM1_ESM.docx]

## **Characterization and measurements techniques**

All the Compounds were characterised by HRTEM, SEM, EDX, CV, EIS, XRD, UV-Vis, and solar simulator to examine their structural, physical, and optical properties. Scanning electron microscope (SEM) analysis was conducted on a Tescan Vega 3, Czech Republic with an acceleration voltage of 10 kV to explore morphological. The structural properties, specifically the crystallinity quality, of all samples were determined through X-ray diffraction (XRD) model Rigaku, Japan instrument analysis using a Cu Kα radiation (λ = 0.154 nm) over the range of 3 - 90 ° (2θ) at a scan speed of 6 °min^-1^. The XRD data was processed and analysed using the FIT2D software. Additionally, the optical properties of the samples were investigated using an ultraviolet-visible spectrometer (UV-Vis) model UV-VIS-NIR spectrophotometer (model V-670, JASCO Co., Ltd., USA. The optical properties were further examined using a). HRTEM was carried out using a FEI Tecnai G2 20 S-Twin with an acceleration voltage of 200 kV. EDX was carried out using (Oxford Instruments EDX) attached with the same SEM instrument for elemental analysis. Electrochemical impedance spectroscopy (EIS), and cyclic voltammetry (CV) can be used to evaluate the electrochemical performance through using an electrochemical workstation (Interface 1010E, Gamry Instruments, USA). Under the condition of AM 1.5-G solar simulator (Enlitech) with an intensity of 100 mW cm^−2^ at a scan rate of 100 mV/s calibrated by NREL standard Si cells, the photocurrent density-voltage (J- V) curves, cell performance of photovoltaic devices were performed in the reverse scan direction (0.0 V to 1.2 V) via 2400 Series Source Measurement Unit (SMU) Instruments meter with the scan speed of 0.1 V/s.

## **Synthesis of single-layer Ti3C2Tx MXene nanosheets**

The MXene has been synthesised at research centre for nanomaterials and energy technology (RCNMET), School of Engineering and Technology, Sunway University [8], [9], [10], [11], [12], [13], [14].

To prepare a Ti_3_C_2_T_x_ MXene sample, the process begins by carefully adding 3 g of Ti_3_AlC_2_ into 30 mL of HF to prevent overheating due to the exothermic nature of the reaction. The solution is stirred at a temperature of 40°C for a duration of 48 hours to allow for etching. Throughout the stirring procedure, Al was etched from the MAX phase material (Ti_3_AlC_2_). Once the etching process is completed, a diluted solution of NaOH is gradually introduced until the pH of the solution reaches 6. The resulting mixture is then filtered and rinsed multiple times with deionized water. Next, the materials undergo treatment in a 0.5 M NaOH solution at room temperature for a period of 3 hours before being filtered again. Repetitions of ultrahigh centrifugation were performed at 3500 rpm (10 min for each cycle) using an ultrahigh centrifuge (Sorvall LYNX 6000, Thermo Scientific). The materials are washed with deionized water and dried overnight at a temperature of 60-70°C. Finally, Ti_3_C_2_T_x_ MXene was obtained by freeze-drying, as shown in figure 2 [15], [16].


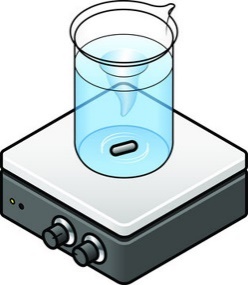

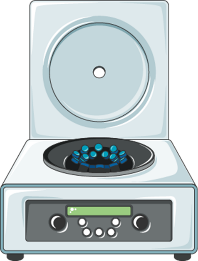

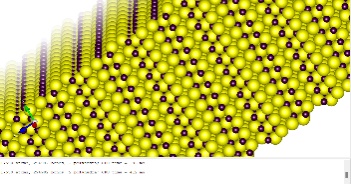

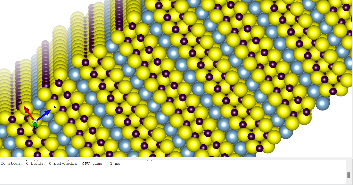

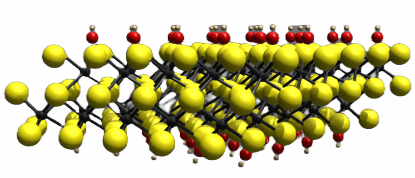


**Etching**

MAX Powder

HCl-LiF Solution

Centrifugation

Etched for 48 hours

**Intercalation**

Single Layer MXene

**Delamination**

**
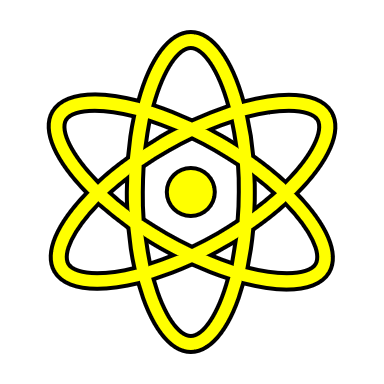
 Ti
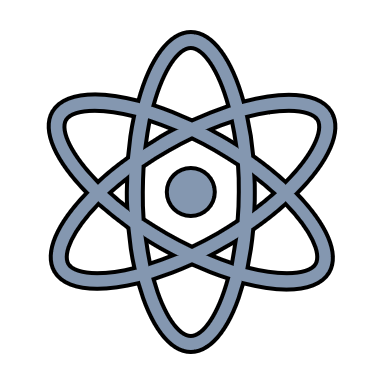
 Al
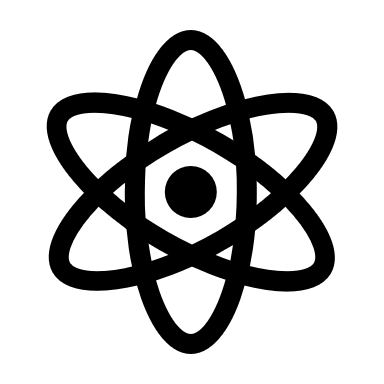
 C**

**
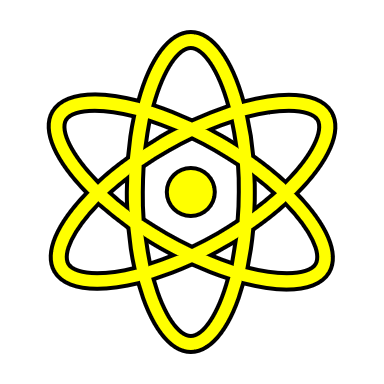
 Ti
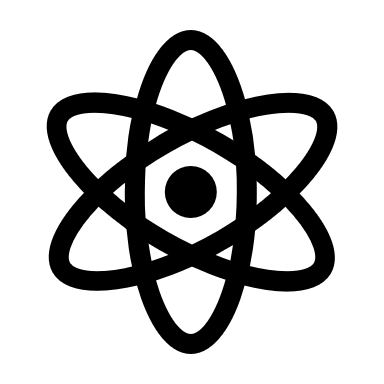
 C**


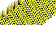


Figure 2: Synthesizing single-layer nanosheets of MXene (2D-Ti_3_C_2_T_x_).

## Structural characterization

To verify the structure of the synthesized Ti_3_C_2_T_x_/CoS, CoS, and Ti_3_C_2_T_x_, SEM and XRD analyses were conducted, and the results are presented in Figure 6 & 7. Prior to the XRD characterization, the samples were dried in a heated oven at 80°C for 18 hours. Subsequently, the black powders were finely ground and carefully placed onto a silica sample holder, flattened using a glass slide. X-ray diffraction was performed to determine the crystalline structures of the MAX phase, Ti_3_C_2_T_x_, CoS, and Ti_3_C_2_T_x_/CoS samples, and the resulting diffraction patterns are shown in Figure 6. The diffraction peaks at 2θ angles of 5.6°, 25.16°, and 47.4° corresponding to the (002), (004), and (110) crystal planes of the Ti_3_C_2_T_x_ [25].

These peaks signify the presence of a tetragonal rutile MXene/CoS nanocomposite structure. Notably, the introduction of the lanthanide dopant does not cause any disruptions, as evidenced by the consistency of the XRD peaks. Moreover, the XRD patterns of nanocomposite, is identical, indicating a homogeneous dispersion within crystal lattice.

Figure 7, spherical CoS nanoparticles can be observed uniformly distributed on Ti_3_C_2_T_x_ flakes, with diameters ranging from 3.54 to 6.69 nm, consistent with the SEM images. Scanning Electron Microscopy (SEM) was used to investigate the micro-structures and morphologies of both Ti_3_C_2_T_x_ and MXene/CoS nanocomposite. The SEM images in Figure 7a shows the according-like structure of Ti_3_C_2_T_x_, which this mechanism facilitates the formation of effective sites, enabling the uniform deposition of CoS nanoparticles onto the conductive MXene sheets. Figure 7b shows without the presence of MXene sheets, only CoS nanoparticles-assembled spheres were observed. SEM images in Figure 7c a depict the MXene/CoS composite, indicating uniform attachment of CoS nanoparticles on the Ti_3_C_2_T_x_ layers.

Figure 6: Powder X-Ray diffraction (XRD) [patterns](https://www.sciencedirect.com/topics/engineering/x-ray-diffraction-pattern) for the pure MXene (Ti_3_C_2_Tx), CoS, and MXene/CoS nanocomposite.


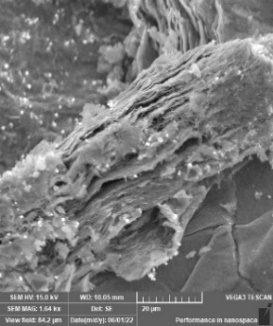

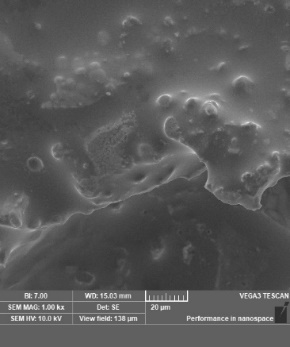

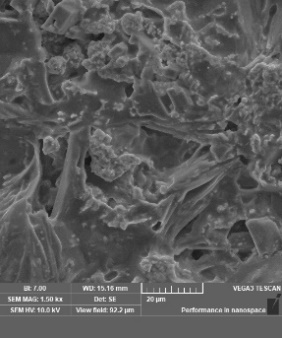


**a**

**c**

**b**

**(MXene) 20 µm**

**(CoS) 20 µm**

**(MXene/CoS) 20 µm**

Figure 7: Structural characterization of the as-synthesized (a) MXene, (b) CoS, and (c) MXene/CoS nanocomposite. The SEM micrographs at two different magnifications.

## Morphological characterization

The morphology of single-layered MXene flakes, Cobalt sulfide, and MXene/CoS nanocomposite were examined using high-resolution transmission electron microscopy (HRTEM) imaging with a JEOL JEM-ARM 200F microscope operating at an accelerating voltage of 200 kV. Around 1 mg of the sample was introduced into a vial containing approximately 4 ml of ethanol. The mixture was placed on a hot plate at 60 °C and stirred using a magnetic stirrer for a duration of 30 minutes. A diluted sample of about 4 μl was taken using a micropipette and deposited onto a carbon-coated copper grid. In TEM analysis, it was observed that the delaminated layers of MXene and CoS nanoparticles exhibited electron beam transparency. Figure 8a displays distinct and well-defined lattice fringes of MXene, showcasing a lattice spacing of 4.22 nm. An HRTEM image of a single-layered Ti_3_C_2_T_x_ flake is presented in figure 8b, providing an overview of the emerging layered structure of MXene. The image demonstrates a thin and electron-transparent morphology, indicating a single-layer structure. The MXene nanosheet is shown to be foldable and flexible, like other MXenes. In HRTEM, the delaminated layers were observed to be electron beam transparent. The selected area electron diffraction of the delaminated MXene image provided a clear indication that the atomic arrangement in the basal planes remains consistent with that of the parent MAX phase. These findings provide further compelling evidence for the conversion of the material from a three-dimensional to a two-dimensional structure. Additionally, the TEM observations indicate no evidence of carbide amorphization.

Significantly, the electron transport layer (ETL) incorporating the MXene/CoS exhibit a substantial enhancement in layer performance. The notable improvement in ETL (electron transport layer) can be primarily attributed to the synergistic effects resulting from the distinctive layered morphology of conductive MXene nanosheets, along with their synergistic cocatalysis with CoS nanoparticles. The HRTEM images presented in figure 8c & d depict small CoS nanoparticles securely anchored onto the framework of Ti_3_C_2_T_x_ MXene sheets, providing visual confirmation of the formation of the Ti_3_C_2_T_x_/CoS composite. The small CoS nanoparticles attached to the MXene nanosheets help shorten electron transport distance. The combination of the layered MXene structure with the presence of small CoS nanoparticles (3.54-6.69 nm) provides a significant number of catalytic active sites. Additionally, the improved mesoporous morphology of the Ti_3_C_2_T_x_/CoS composite contributes to a high specific surface area and facilitates rapid electron transfer channels. Consequently, the Ti_3_C_2_T_x_/CoS nanocomposite demonstrates great potential as an effective material for enhancing the photovoltaic performance of electron transport layer (ETL). However, with the addition of a small quantity of nanocomposite into the SnO_2_ lattice, a pinhole-free surface morphology of SnO_2_ was observed in figure 8e, f, & g. It is important to confirm the formation of a hexagonal lattice characteristic of Ti_3_C_2_T_x_ MXene, as well as diffraction rings attributed to CoS. This suggests that the addition of nanocomposite effectively improves the coverage quality of the SnO_2_ layer and reduces surface-state traps, enhancing the collection ability of photo-generated electrons. It is important to optimize the concentration of nanocomposite [26]. Line profiles of cobalt sulfide and SnO_2_- MXenes/CoS nanocomposite ETL also shown in figure 8h &i; d-spacing between adjacent lattice planes of 0.2099 and 0.333 nm, respectively. This demonstrates that the incorporation of MXenes increases the lattice spacing facets of SnO_2_ without altering the crystal's existence.




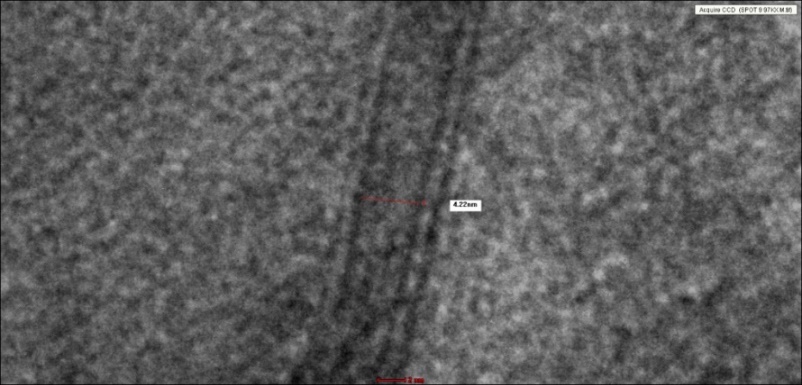

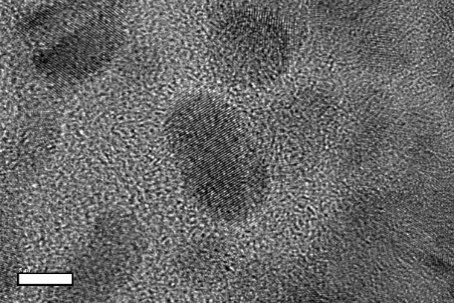


1. **MXene**
2. **MXene**
3. **CoS**


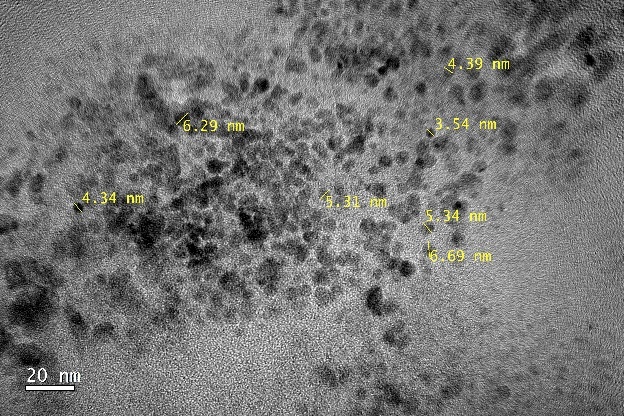

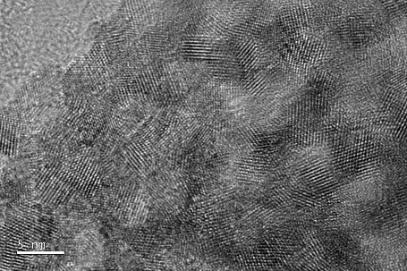


1. **CoS**
2. **MXene-CoS**


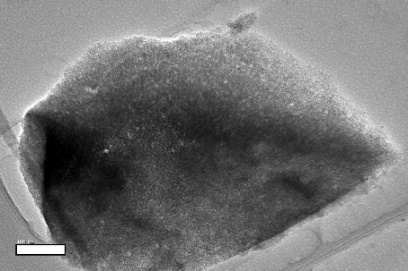

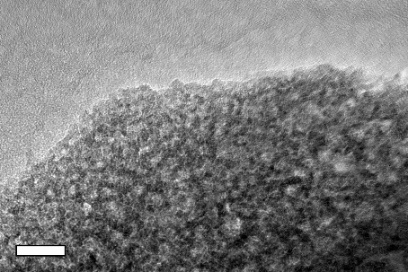


1. **MXene-CoS**
2. **MXene-CoS**


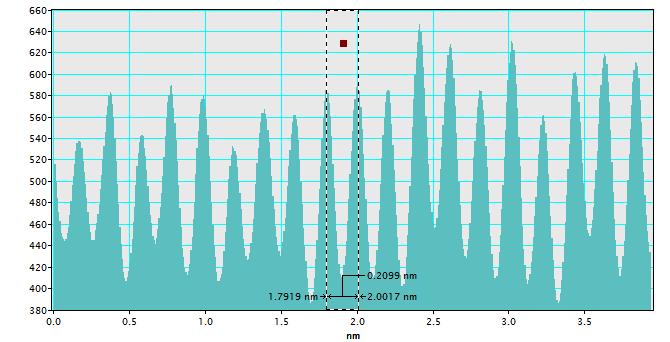

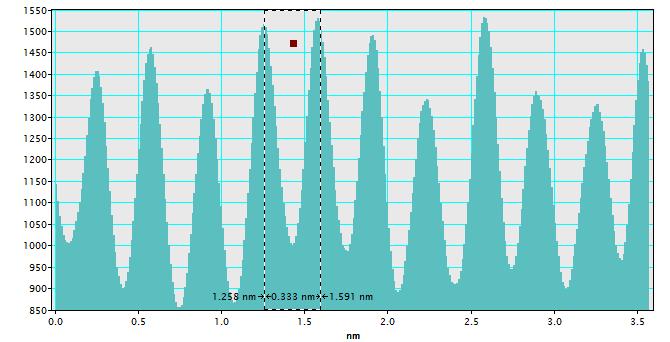


**(h) CoS**

1. **MXene-CoS**

Figure 8: Top view of typical bright-field HRTEM images with different magnification and contrast intensity profile indicates the lattice parameter of the (a-b) single layered MXene (Ti_3_C_2_T_x_) flake, (c,d,h) Cobalt sulfide, and (e-g,i) MXene-CoS.

The nanocomposite-ETL demonstrated superior crystallinity with higher quality and effective carrier transport. The composition of the MXene also contributes to the improvement of the crystalline quality and optical properties of the SnO_2_ layer. The introduction of nanocomposite into the tin precursor solution reduces the aggregation of SnO_2_ crystals, resulting in a surface morphology without pinholes. This, in turn, enhances the conductivity and transparency of SnO_2_, facilitating charge injection and electron transfer.

## Energy dispersive X-ray spectroscopy

Elemental map imaging and spatial distribution of all elements were verified using energy dispersive X-ray spectroscopy (EDX) with an Oxford Instrument. Spot analysis was conducted to determine quantified distribution of elements in various sections of the MXene, and MXene/CoS nanocomposite. The EDX findings of the single-layered MXene nanosheets demonstrated the presence of C, Ti, and minor amounts of O and F on the MXene surface, along with a very small quantity of Al, as depicted in figure 9a.


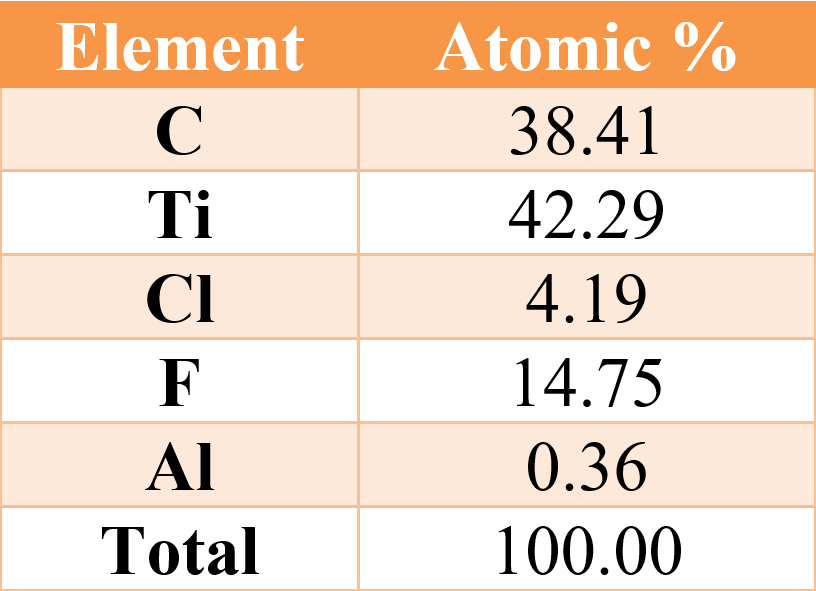
(a)
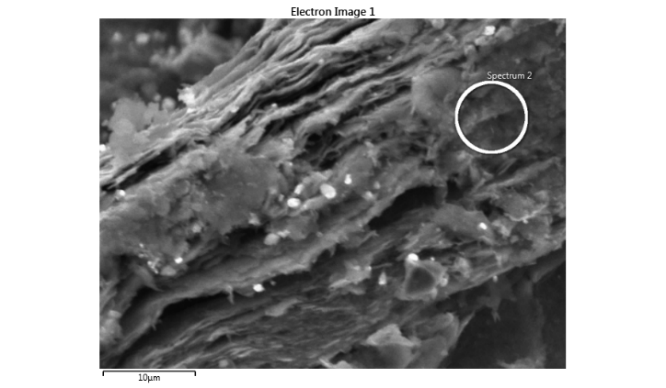

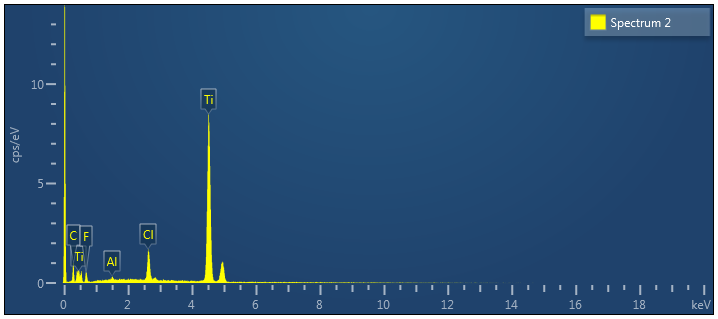


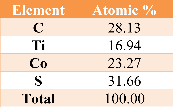
 (b)
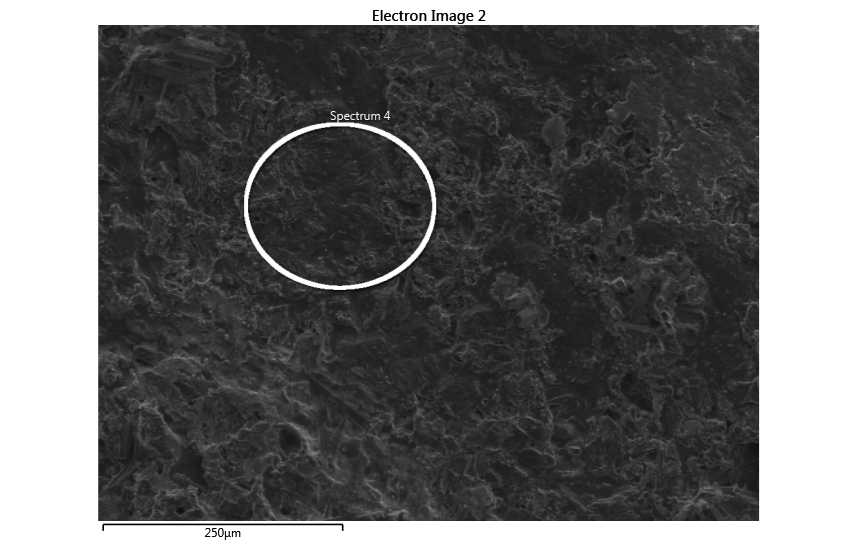

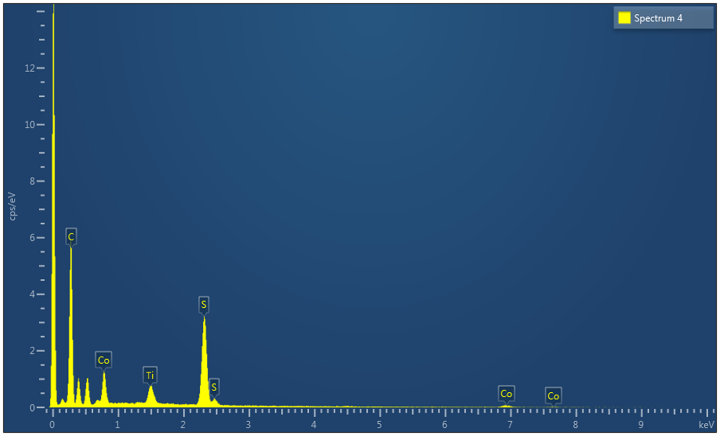


Figure 9: SEM and EDX Analysis of MXene, Mineral components of the (a) MXene and (b) nanocomposite Samples No., Element Weight (%), Atomic (%).

Furthermore, the EDX analysis of the MXene/CoS nanocomposite revealed the presence of titanium (Ti), carbon (C), cobalt (Co), and sulfur (S) on the surface of sample, as illustrated in figure 9b. The absence of specific elements like OH, F, and O in the MXene component of the nanocomposite was intentional and controlled through synthesis and processing steps to optimise the nanocomposite's properties.
